# Association of magnitude of weight loss and weight variability with mortality and major cardiovascular events among individuals with type 2 diabetes mellitus: a systematic review and meta-analysis

**DOI:** 10.1186/s12933-022-01503-x

**Published:** 2022-05-16

**Authors:** Shan Huang, Ke Shi, Yan Ren, Jin Wang, Wei-Feng Yan, Wen-Lei Qian, Zhi-Gang Yang, Yuan Li

**Affiliations:** 1 Department of Radiology, West China Hospital, Sichuan University, 37# Guo Xue Xiang, Chengdu, 610041 Sichuan China; 2 Department of Endocrinology and Metabolism, West China Hospital, Sichuan University, 37# Guo Xue Xiang, Chengdu, 610041 Sichuan China; 3 Department of Radiology, State Key Laboratory of Biotherapy, West China Hospital, Sichuan University, No.37 Guoxue Xiang, Chengdu, 610041 China

**Keywords:** Type 2 diabetes mellitus, Weight loss, Weight variability, Mortality, Major cardiovascular events

## Abstract

**Background:**

Weight management is strongly promoted for overweight or obese patients with type 2 diabetes (T2DM) by current guidelines. However, the prognostic impact of weight loss achieved without behavioural intervention on the mortality and cardiovascular (CV) outcomes in diabetic patients is still contested.

**Methods:**

We searched the PubMed, Embase, and Cochrane Library databases for studies that investigated the association of weight loss or weight variability with mortality and CV outcomes. Results of studies that measured weight loss by percentage weight loss from baseline and stratified it as > 10% and 5–10% or studies that computed weight variability were pooled using random effects model. Study quality was evaluated using the Newcastle–Ottawa Scale.

**Results:**

Thirty eligible studies were included in the systematic review and 13 of these were included in the meta-analysis. Large weight loss (> 10%) was associated with increased risk of all-cause mortality (pooled hazard ratio (HR) 2.27, 95% CI 1.51–3.42), composite of major CV events (pooled HR 1.71, 95% CI 1.38–2.12) and CV mortality (pooled HR 1.50, 95% CI 1.27–1.76) among T2DM patients. Moderate weight loss showed no significant association with all-cause mortality (pooled HR 1.17, 95% CI 0.97–1.41) or CV outcomes (pooled HR 1.12, 95% CI 0.94–1.33). Weight variability was associated with high hazard of all-cause mortality (pooled HR 1.54, 95% CI 1.52–1.56).

**Conclusions:**

Large weight loss and large fluctuations in weight are potential markers of increased risk of mortality and CV events in T2DM patients. Maintaining a stable weight may have positive impact in these patients.

**Supplementary Information:**

The online version contains supplementary material available at 10.1186/s12933-022-01503-x.

## Background

Weight management is strongly promoted for overweight or obese patients with type 2 diabetes (T2DM) by current guidelines to reduce the risk of diabetes complications and improve outcomes [1]. Substantial weight loss achieved after bariatric surgery in severely obese T2DM patients has been shown to reduce the risk of mortality and cardiovascular (CV) events [2]. Studies have also shown that intentional weight loss can lead to diabetes remission [3] and improvement in blood pressure and lipid control [4, 5]. Moreover, according to a post-hoc analysis of a large prospective clinical trial, > 10% weight loss after intensive lifestyle intervention was associated with lower CV event rate among overweight T2DM participants [6]. Moderate loss of body weight in the year after diabetes diagnosis was also found to be associated with a lower risk of CV events at 10 years [7].

However, multiple studies [8–12] have found no effect or adverse effect of weight loss on all-cause mortality and major CV events in patients with T2DM. Weight loss is a common symptom of T2DM which is attributable to aggravated protein catabolism and muscle oxidative damage induced by hyperglycemia and hyperinsulinemia [13]. Large unintentional weight loss is indicative of loss of bone and muscle tissues and is associated with increased risk of mortality and CV events [14–16].

A previous systematic review of studies published before July 2019 found that weight gain is associated with increased risk of cardiovascular disease (CVD) and mortality, whereas the effect of weight loss on mortality and CV outcomes was not clear [17]. We performed an updated systematic review and meta-analysis by including additional relevant studies published in the recent 2 years. The objective was to synthesise the available evidence on the effect of weight loss achieved without behavioural intervention on the all-cause mortality and major CV events in patients with T2DM.

## Methods

This study was conducted in accordance with the PRISMA (preferred reporting items for systematic reviews and meta-analysis) statement. It was registered with PROSPERO (Number CRD42022301756).

### Search strategy and study selection

A comprehensive literature search was performed in the PubMed, Embase, and Cochrane Library databases to identify all relevant articles published from January 2000 to February, 2022. During the revision process, we re-searched the databases using a modified search strategy. The reference lists of the included studies and relevant review articles were manually screened to identify other potential studies.

The search strategy employed a combination of Mesh and text words using the following three sets of terms: (1) “diabetes mellitus”; (2) “weight loss”, “weight change”, or “weight variability”; and (3) outcome-related terms, such as: “mortality”, “cardiovascular events”. Customised strategies for different databases were adapted where necessary. Details of the search strategy are presented in the Additional file 1.

The inclusion criteria for screened full-text articles were: (1) observational cohort studies or post hoc analyses of clinical trials of adult patients with T2DM (aged ≥ 18); (2) investigated the association of weight loss or weight variability with long-term outcomes; and (3) adjusted hazard ratios (HR) or relative risks (RR) with 95% confidence intervals were reported for all-cause mortality, CV mortality and a composite of major CV events. Two reviewers (KS and YR) independently assessed the eligibility of the studies that qualified the inclusion criteria. Disagreements, if any, were resolved by consensus. If two or more studies based on the same study cohort reported data for different outcomes, they were included and analysed for specific outcomes.

### Data extraction and assessment for study quality

Two reviewers (WFY and WLQ) independently extracted the data pertaining to the following variables: first author, year of publication, original dataset, region/country, study type, year of enrolment, number of participants and proportion of female subjects, age, diabetes duration, baseline CVD exclusion, intervention, assessment of body weight, weight change interval, follow-up duration, and the study outcomes.

Two reviewers (JW and YL) independently evaluated the quality of included studies by using the Newcastle–Ottawa scale. Disagreements were resolved by discussion and further review. The quality of the studies was graded as poor (< 4 points), fair (4–6 points), or good (> 7 points).

### Statistical analysis

The primary outcome of interest was all-cause mortality. The secondary outcome was a composite of major CV events and CV mortality. Since we aimed to assess the effect of weight loss achieved without behavioural intervention on the outcomes, for studies that included behavioural intervention, only data pertaining to the control group was pooled. Weight loss more than 10% from baseline was defined as major or large weight loss. Weight loss of 5–10% was defined as moderate weight loss. Studies with unavailable data on a specific endpoint were excluded from the pooled analysis for that endpoint. We collected available HRs or RRs after full adjustment as originally reported in each study. The list of confounding variables that were adjusted for in each study is provided in the Additional file 1. We pooled the logHRs using random effects model considering the underlying heterogeneity across studies. Sensitivity analysis was performed to evaluate the influence of individual studies on the results of the meta-analysis. I^2^ statistics was used to assess heterogeneity. An I^2^ value of > 50% was considered indicative of statistically significant heterogeneity. All statistical analyses were conducted using STATA version 15.0 (Stata Corp, College Station, TX).

## Results

### Study selection

**Fig. 1:**
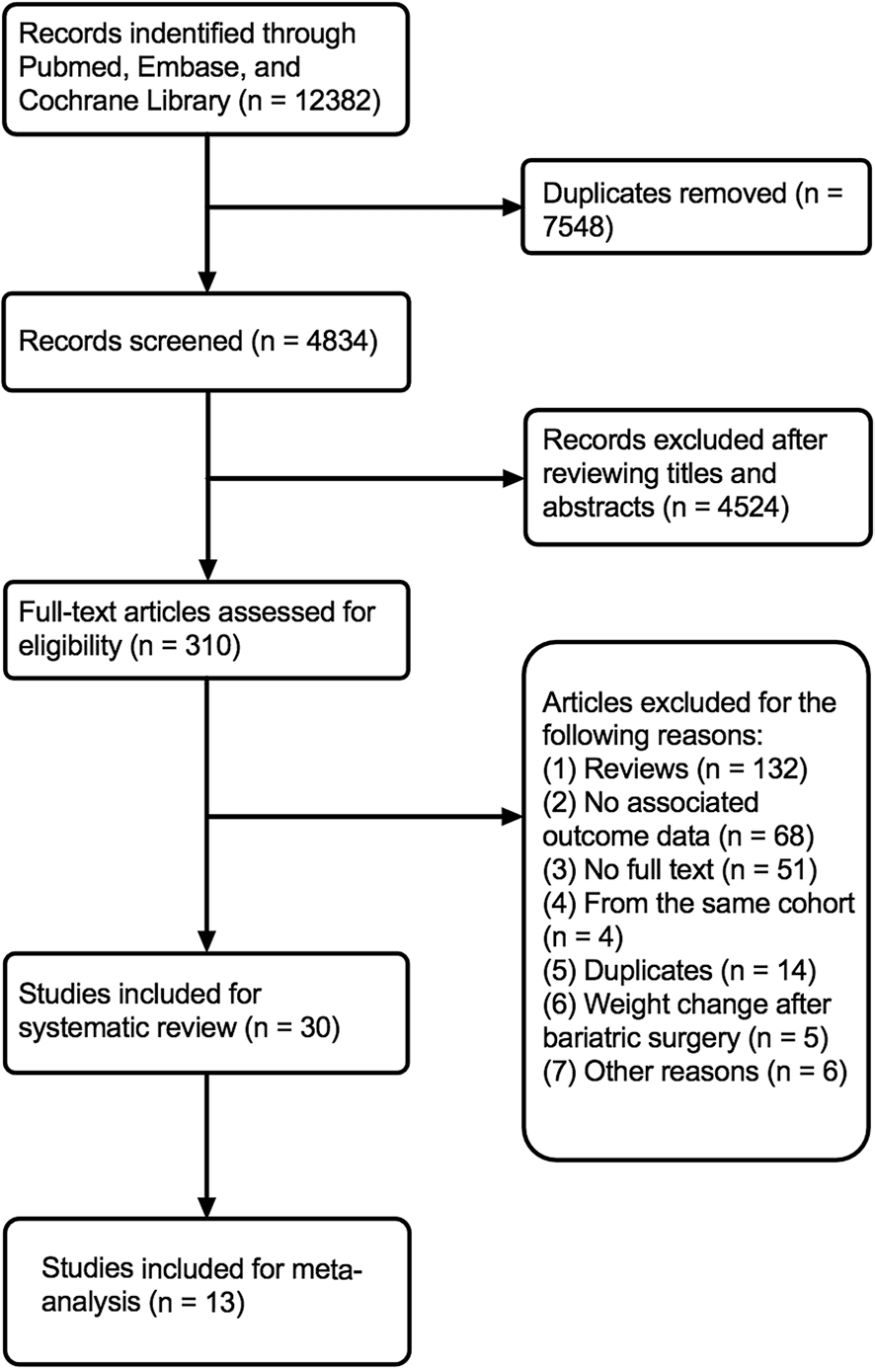
The flow chart of study selection

A schematic illustration of the literature search process and study selection is shown in Fig. 1. The initial search of databases yielded 12,382 articles. After removing duplicates and screening the titles and abstracts, 310 full-text articles were assessed for eligibility. As a result, 30 studies were included in the systematic review. Data from 7 studies and 6 studies were pooled for assessment of the prognostic impact of weight loss and weight variability, respectively. Studies were excluded from the pooled analyses mainly due to the use of different weight change unit or cutoffs for weight change magnitude.

### Characteristics of the included studies

Characteristics of studies included in this systematic review and meta-analysis are presented in Table 1 and Additional file 2: Table S1. Fourteen studies [6–8, 10, 12, 15, 18–26] were post-hoc analysis of prospective clinical trials. The sample size in the included studies ranged from 230 to 1,522,241. Average age of the included participants was 59.5 years, ranged from 53.6 to 66 years. One study cohort [11] consisted of 77.8% of females. Ninety-seven percent of another study cohort [27] was male. The proportion of female participants in other studies ranged from 31.7 to 62.0%. Eight studies [7, 9, 11, 12, 22, 26–28] included patients with newly-diagnosed T2DM. Four studies [6, 23, 29, 30] included only overweight or obese T2DM patients. Ten studies [9, 11, 18, 22, 24, 28, 31–34] excluded individuals with pre-existing CV comorbidities. One study [19] included T2DM patients with a recent acute coronary syndrome. Ten studies [8, 9, 11, 12, 18, 20–23, 35] used a more than 2-year weight change interval to make sure that the change in weight was sustained. The follow-up time ranged from median 1.6 years (IQR: 1.0–2.1) to up to 26 years. In 19 studies [6–9, 11, 12, 18, 20–22, 26, 27, 30, 32, 35–37], the median or mean follow-up time was of > 5 years. Other three studies [11, 23, 38] reported a maximum follow-up time more than 10 years.

**Table 1:** Characteristics of studies included in the meta-analysis

Study	Study name or dataset	Country/region	Study design	Year of enrollment	Diabetes duration	Baseline CVD excluded	Intervention	No. patients (% female)	Age (years)	Weight change measurement	Weight change interval	Follow-up	Event for analysis
Lee et al. 2020	ADVANCE	20 countries	Prospective	2001–2003	Mean 7.84 years	No	intensive glucose lowering, standard glucose control, blood pressure lowering, placebo	10,081 (42.2%)	66	Percentage weight change from baseline (%)	2 years	Median 3.0 years during ADVANCE and 8.4 years for ADVANCE-ON (n = 8064)	All-cause mortality, CV mortality, major macrovascular events, microvascular events
Kim et al. 2019	KNHIS	Korea	Retrospective	2007–2012	Newly-diagnosed	Yes	No	173,246 (34.6)	55.8	Percentage weight change from baseline (%)	2 years	Median 5.5 years	All-cause mortality, Stroke, MI
Gregg et al. 2016	Look AHEAD	16 clinical sites in the USA	Prospective	2001.8–2004.4	Mean 6.78 years	No	intensive lifestyle intervention, diabetes support and education	4834 (60.2)	59.1	Percentage weight change from baseline (%)	1 year	Median 10.2 years (IQR 9·5–10.7)	composite of CV events
Doehner et al. 2012	PROactive study	Germany	Prospective	2001–2002	Mean 8 years	No	pioglitazone	5202 (33.9)	62 ± 8	Percentage weight change from baseline (%)	1 year	Mean 34.5 months	All-cause mortality, CV mortality
Ferreira et al. 2021	EXAMINE trial	France	Prospective	2009	Mean 7.2 years	No	Alogliptin, placebo	5380 (32.1)	60.8	Percentage weight change from baseline (%)	Median 1.6 (IQR 1.0–2.1) years	Median 1.6 (IQR 1.0–2.1) years	All-cause death, CV death, CVD/HFH, Primary outcome
Hu et al. 2021	NHS and HPFS	USA	Retrospective	1980 and 1986	Newly-diagnosed	Yes	No	11,262 (77.8)	female: 62.4 ± 9.2 male: 63.2 ± 8.8	Percentage weight change from baseline (%)	2 years	Up to 26 years	All-cause mortality, CV mortality, Cancer mortality, Deaths from other causes
Strelitz et al. 2019	ADDITION-Europe trial	Europe	Prospective	2001–2006	Newly-diagnosed	No	multifactorial treatment and routine care	2730 (42)	60.2 ± 6.9	Percentage weight change from baseline (%)	5 years	Mean 5 ± 1 years	All-cause mortality, CVD
Nam et al. 2020	KNHIS	Korea	Retrospective	2009.1–2010.12	NA	Yes	No	624,237 (34.1)	56.8 ± 11.7	SD, coefficient of variation, BIM, ASV	5 years	Mean 7.7 years	all-cause mortality, MI, stroke
Bangalore et al. 2018	CARDS, ASPEN, TNT trial	16 countries	Prospective	1996–2001	NA	Yes	atorvastatin	6408 (31.7)	61.7 ± 8.1	ASV	Throughout the whole follow-up time	CARDS: median 3.9 years, ASPEN: median 4 years, TNT: median 4.9 years	Coronary event, Major coronary event, CV event, Death, CV death, MI, stroke
Yeboah et al. 2019	ACCORD study	USA and Canada	Prospective	2001–2005	Mean 10.8 ± 7.6 years	No	intensive blood pressure, glycemic, lipid treatment, and standard care	10,251 (38.5)	62.8 ± 6.6	ASV, weight change(kg)	Mean 3.7 years (up to 7 years)	Mean 3.7 years (up to 7 years)	Primary outcome, CHF, total death, microvascular event
Ceriello et al. 2021	Swedish National Diabetes Register	Sweden	Retrospective	2000.1–2019.9	< 5 years: 71,413 (71%), 5–10 years: 15,012 (14.9%); > 10 years: 14,126 (14%)	Yes	No	100,576 (44.3)	64.2	SD	3 years	Median 4.4 (IQR 2.1–6.7)	Primary composite outcome, All-cause mortality, MI, Stroke
Aucott et al. 2016	SCI-DC	Scotland	Retrospective	2002–2006	Newly-diagnosed	No	No	29,316 (45.6)	58.4 ± 12	coefficient of variation	2 years	Median 5.2 (IQR 3.8–6.2)	All-cause mortality, MI, CHF
Kaze et al. 2022	Look AHEAD	16 clinical sites in the USA	Prospective	2001–2004	mean 4.9 years	No	diabetes support and education	1775(62)	Mean 58.5 years	coefficient of variation, SD	3 years	median6.7 (IQR 6.0–7.4)	All-cause mortality, CV mortality, CV events

ADVANCE, The Action in Diabetes and Vascular disease: preterAx and diamicroN-MR Controlled Evaluation; KNHIS, Korean National Health Insurance System; Look AHEAD, Action for Health in Diabetes; PROactive, PROspective pioglitAzone Clinical Trial In macroVascular Events; EXAMINE, cardiovascular outcomes study of alogliptin in patients with type 2 diabetes and acute coronary syndrome; NHS, Nurses’ Health Study; HFPS, health professionals follow-up study; ADDITION, Anglo–Danish–Dutch study of intensive treatment in people with screen-detected diabetes in primary care; CARDS, collaborative atorvastatin diabetes study; ASPEN, atorvastatin study for prevention of coronary heart disease endpoints in non-insulin-dependent diabetes mellitus; TNT, treating to new targets; ACCORD, action to control cardiovascular risk in diabetes; SCI-DC, Scottish care information diabetes collaboration; CVD, cardiovascular disease; MI, myocardial infarction; CVD/HFH, composite of cardiovascular death or heart failure hospitalization; CHF, congestive heart failure

The studies investigated the associations of weight change with mortality and CV events using various parameters [such as, body mass index (BMI), kg, pounds, %weight change, etc.,] and different cutoff levels to categorise the degree of weight change. We pooled the outcomes of studies using cutoffs of 5–10% and > 10% for weight loss. Seven studies [24–26, 32, 33, 36, 37] reported the association of weight variability with mortality and CV events. Among them, weight variability was assessed using average successive variability (ASV), coefficient of variation, and standard deviation (SD).

According to the Newcastle–Ottawa quality assessment scale, 20 studies were graded as having good quality and 10 studies were graded as having fair quality. Details of quality evaluation are listed in Additional file 2: Table S2.

### Association between weight loss and all-cause mortality

Among the 18 studies that investigated the association between weight loss and risk of all-cause mortality (outcomes are presented in Additional file 2: Table S3), 14 studies[8–12, 19–21, 23, 27, 29, 32, 35, 38] found that weight loss was associated with an increased risk of all-cause mortality (HR [95% CI] ranging from 1.21 [1.03–1.41] to 5.6 [3.96–7.91]). Other 3 studies [7, 28, 31] found no association between weight loss and mortality. Only the study by Williamson et al. [30] reported a reduced risk of mortality among participants with intentional weight loss (HR 0.75, 95% CI 0.67–0.84).

**Fig. 2:**
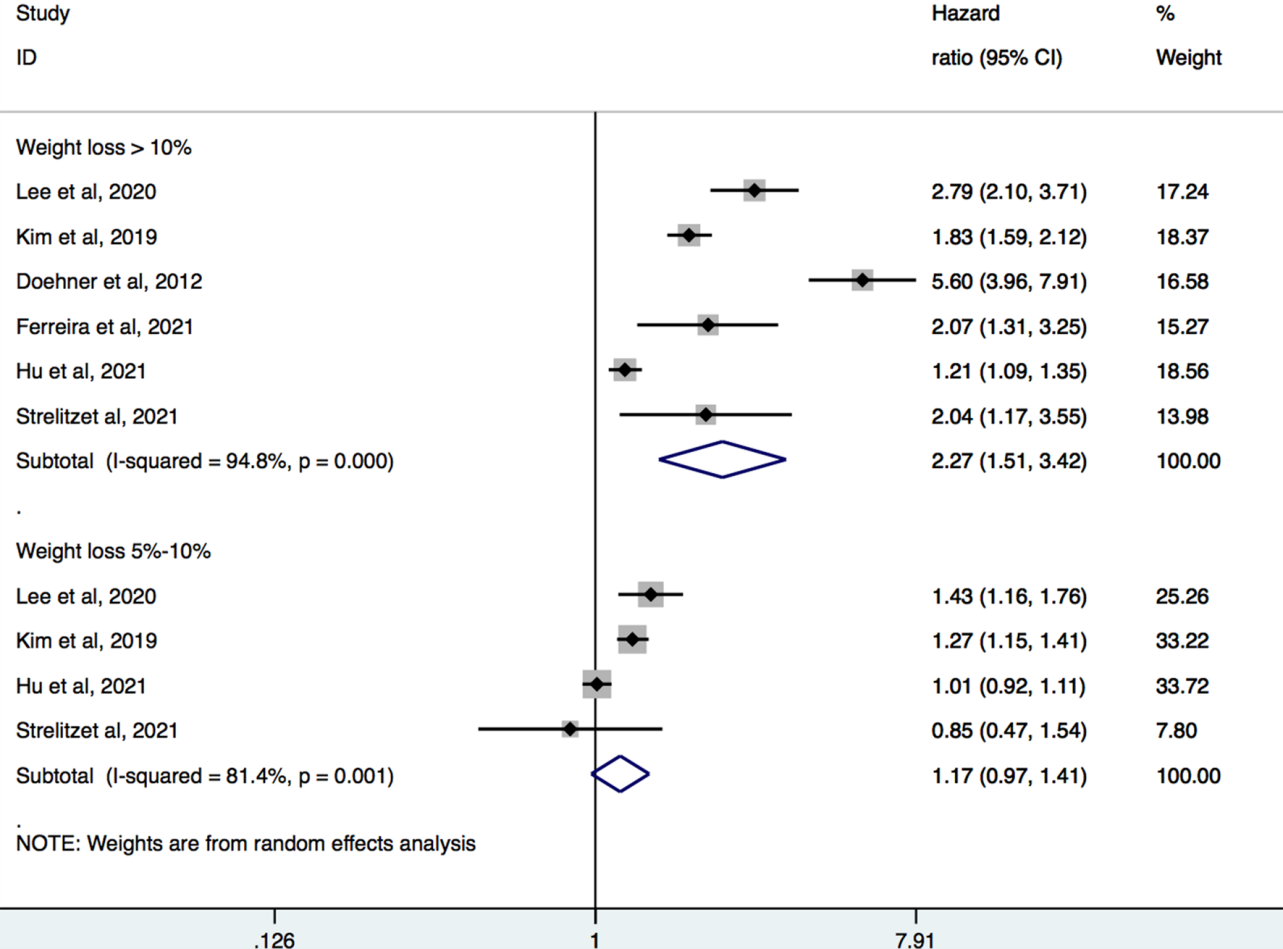
Forest plot of hazard ratios of all-cause mortality for large and moderate weight loss

Six studies [8–12, 19] with a total of 207,901 participants were pooled for the analysis of relation between magnitude of weight loss and all-cause mortality (Fig. 2). Meta-analysis showed that large weight loss (> 10%) was associated with increased hazard of all-cause mortality (pooled HR 2.27, 95% CI 1.51–3.42, I^2^ = 94.8%). There was no significant association between moderate weight loss (5–10%) and risk of all-cause mortality (HR 1.17, 95% CI 0.97–1.41, I^2^ = 81.4%).

Sensitivity analysis revealed stability of the primary results after exclusion of any one of the included studies (Additional file 2: Fig. S1). Subgroup analysis based on diabetes history showed that among participants with large weight loss, those with established T2DM at baseline had higher risk of all-cause mortality (pooled HR 3.22, 95% CI 1.86–5.58) compared to those with newly-diagnosed T2DM (pooled HR 1.58, 95% CI 1.11–2.26) (Additional file 2: Fig. S2). In studies that excluded participants with CVD risk profile at baseline, large weight loss was still associated with mortality (pooled HR 1.55, 95% CI 1.09–2.19) (Additional file 2: Fig. S3).

### Association between weight loss and cardiovascular events

Eight studies reported the association between weight loss and a composite of CVD events (outcomes are presented in Additional file 2: Table S4). Three of them [8, 19, 31] found that weight loss was associated with composite of CVD events (HR [95% CI] ranging from 1.31 [1.02–1.68] to 2.14 [1.15–1.86]). In the ADDITION-Cambridge trial [7], moderate weight loss was associated with a decreased risk of CVD events for (HR 0.52, 95% CI 0.32–0.86). The other studies [6, 18, 20, 23] found no significant association between weight loss and CVD events. In addition, two studies [32, 39] showed that weight loss was related to increased risk of myocardial infarction (MI) (HR [95% CI] 1.18 [1.13–1.24] and 1.39 [1.21–1.60]), stroke (HR [95% CI] 1.09 [1.03–1.14] and 1.50 [1.33–1.70]), and heart failure (HR [95% CI]: 1.64 [1.50–1.79]). One study [35] found that both moderate (5–10%, HR [95% CI] 1.09 [1.06–1.13]) and large weight loss (> 10%, HR [95% CI] 1.24 [1.17–1.31]) were associated with new-onset atrial fibrillation (AF). In contrast, the study by Chan et al. [40] observed that weight loss > 5% following sodium–glucose cotransporter 2 inhibitor treatment was related to a lower risk of incident AF (HR [95% CI] 0.39[0.22–0.68]). Another study [34] did not find a significant association between relative weight loss (> 1 BMI unit) and incident AF.

Eight studies analyzed the relationship between weight loss and CV mortality. Four of these studies [8, 11, 19, 20] reported an increased risk of CV mortality (HR [95% CI] ranging from 1.175 [1.020–1.353] to 2.76 [1.87–4.09]). The other 3 studies [21, 23, 28] found no significant association between weight loss and CV mortality. Only the study by Williamson et al. [30] reported a protective effect of intentional weight loss on CV mortality (HR = 0.72, 95% CI [0.63–0.82]).

**Fig. 3:**
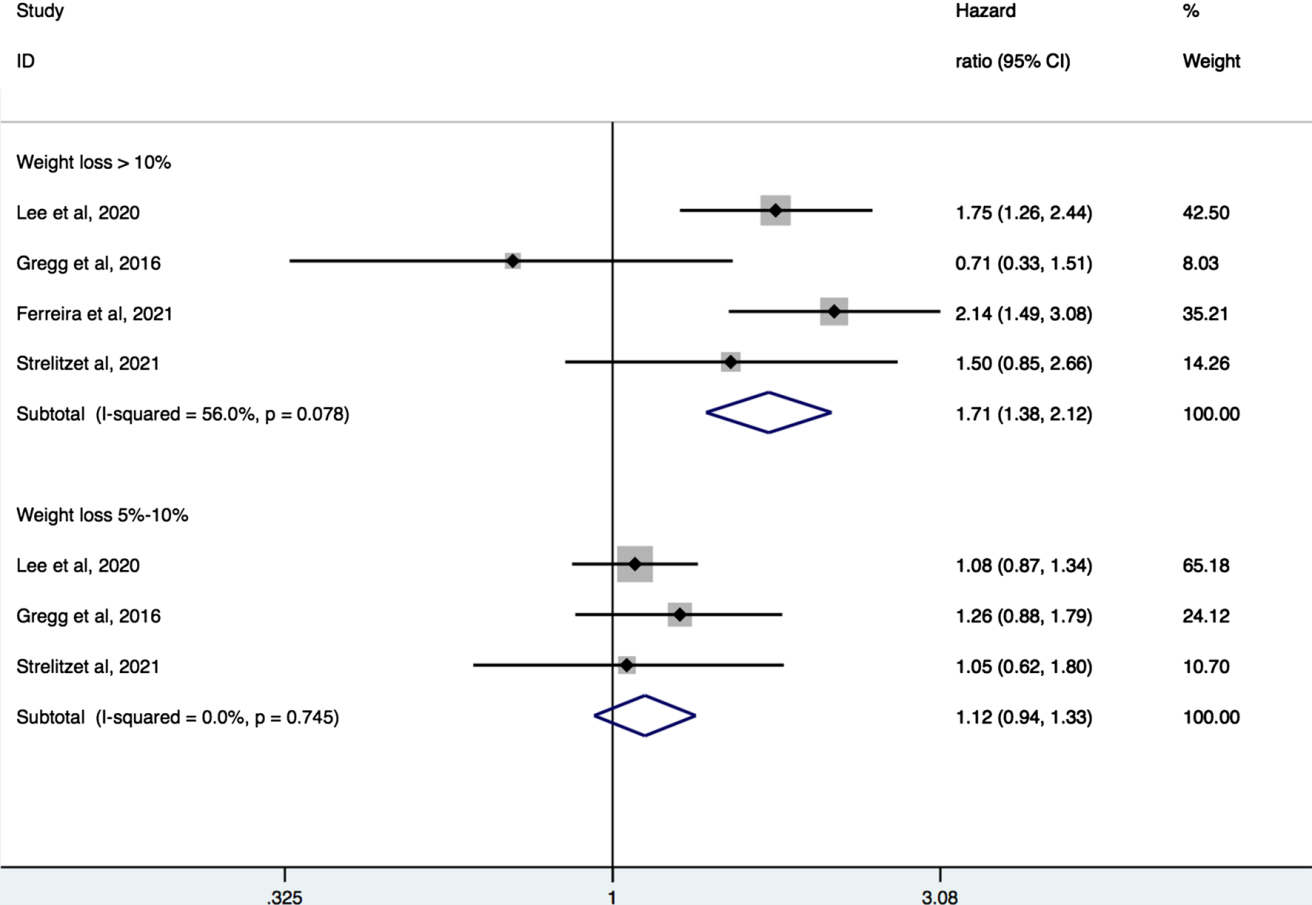
Forest plot of hazard ratios of major cardiovascular events for large and moderate weight loss

**Fig. 4:**
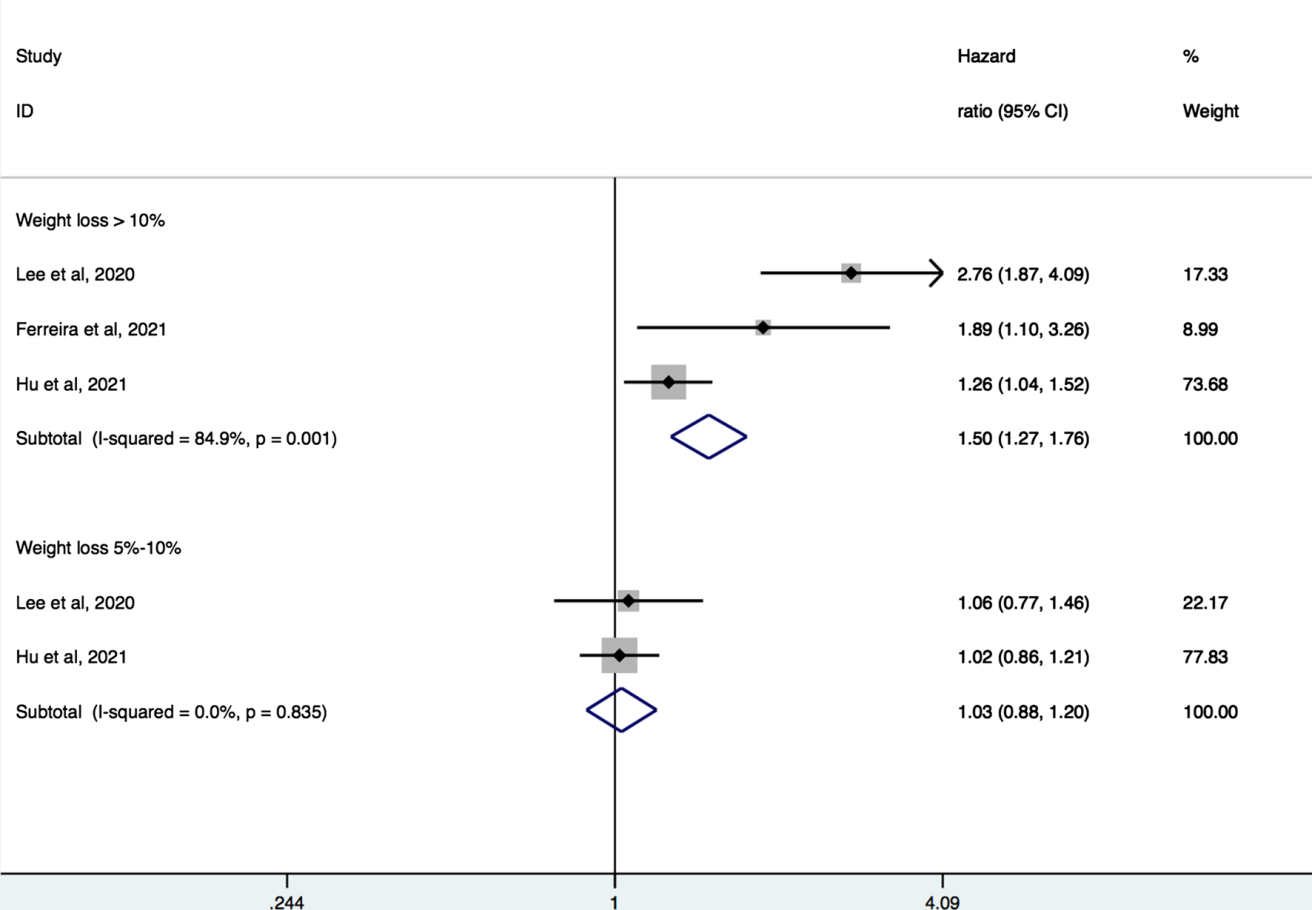
Forest plot of hazard ratios of cardiovascular mortality for large and moderate weight loss

Four studies [6, 8, 12, 19] with a total of 20,550 participants were pooled for the analysis of relation between magnitude of weight loss and CVD events. Meta-analysis showed that large weight loss (> 10%) was associated with increased hazard of composite CV events (pooled HR 1.71, 95% CI 1.38–2.12, I^2^ = 56%) and CV mortality (pooled HR 1.50, 95% CI 1.27–1.76, I^2^ = 84.9%). There was no significant association of moderate weight loss (5–10%) with composite of CV events (pooled HR 1.12, 95% CI 0.94–1.33, I^2^ = 0%) or CV mortality (pooled HR 1.03, 95% CI 0.88–1.20, I^2^ = 0%) (Figs. 3 and 4).

### Interactions between age, sex, baseline BMI and weight loss on outcomes

Six studies [8, 9, 12, 18, 22, 29] analysed the interaction between weight loss and baseline age (> 65 years vs < 65 years or > 60 years vs < 60 years). None of these studies found a significant difference in association between weight loss and outcomes (*P*-for-interaction > 0.05 for all).

Three studies [9, 18, 29] stratified the outcomes by sex. No significant difference was found in the risk of mortality or CV events between men and women (*P*-for-interaction > 0.05 for all). In the study by Wedick et al. [38], weight loss over 10 pounds was associated with increased risk of all-cause mortality only in diabetic men, not in women (HR 3.85, 95% CI [2.15–6.24] vs. HR 1.58, 95% CI [0.70–3.87]). The Nurses’ Health study cohort [11] (almost 80% were women) had a HR [95% CI] of 1.27 [1.11–1.46] for all-cause mortality and 1.46 [1.15–1.86] for CV mortality among those who lost more than 10% of weight. The Veterans Health Administration study cohort [27] (97% were men) had a HR [95% CI] of 1.43 [1.33–1.53] for all-cause mortality among those who lost more than 5% of weight.

Four studies [9, 10, 19, 20] observed that participants with higher BMI (overweight or obese) at baseline had reduced risk of mortality or CV outcomes, while normal weight or underweight participants had increased risk of these outcomes. No difference was observed in the association of weight loss and outcomes when stratified by baseline BMI (BMI < 25 vs BMI > 25 or BMI < 30 vs BMI > 30, *P*-for-interaction > 0.05 for all)[8, 9, 12, 18, 19, 29].

### Association between weight variability and outcomes

**Fig. 5:**
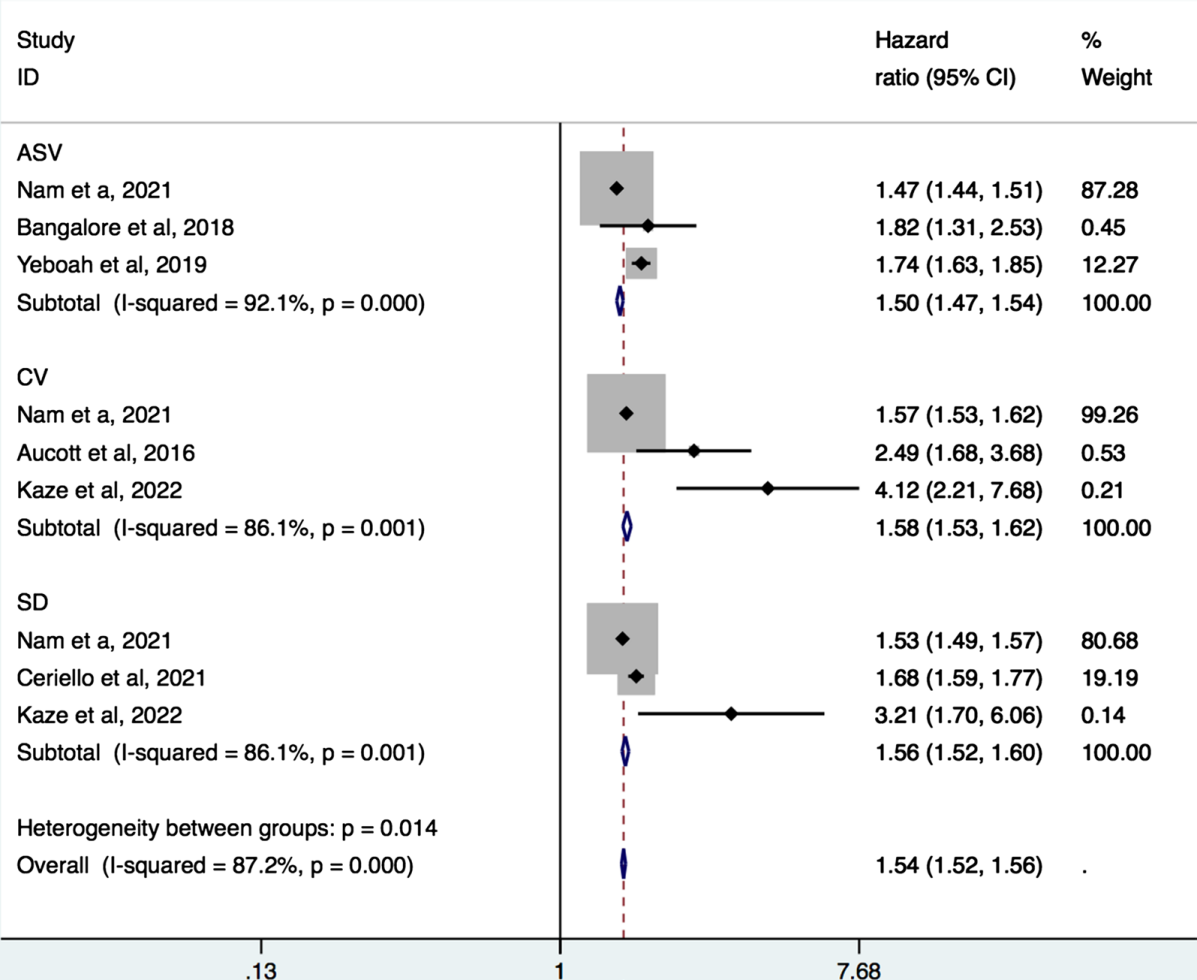
Forest plot of hazard ratios of all-cause mortality for weight variability. ASV, average successive variability; CV, coefficient of variation; SD, standard deviation

In 6 studies [24–26, 32, 33, 36] with a combined study population of 772,563, fluctuation in body weight was associated with increased all-cause mortality and major CV outcomes among T2DM patients. Another study [37] reported that high weight variability was associated with incident AF (HR [95% CI] 1.16 [1.12–1.20]). The pooled analyses showed that ASV (pooled HR 1.50, 95% CI 1.47–1.54), coefficient of variation (pooled HR 1.58, 95% CI 1.53–1.62), and SD (pooled HR 1.56, 95% CI 1.52–1.60) were found to predict increased risk of all-cause mortality, respectively and in total (pooled HR 1.54, 95% CI 1.52–1.56) (Fig. 5).

## Discussion

The major findings of this systematic review and meta-analysis are as follows. First, synthesis of observational studies revealed the association of large weight loss (> 10%) with increased risk of all-cause mortality, a composite of major CV events and CV mortality. Second, moderate weight loss (5–10%) was not associated with all-cause mortality or CV events. In addition, large fluctuation in body weight was also associated with increased risk of all-cause mortality.

### Impact of intentional or unintentional weight loss on outcomes

Despite the significant heterogeneity among the studies that investigated the effect of weight loss by intentionality, the consensus was that unintentional weight loss is markedly related to higher death rate. We were unable to determine whether the weight loss was intentional or unintentional in most of the studies. However, in a majority of the included studies, behavioral interventions were not administered during the trials. And we only incorporated the data pertaining to the control group that received diabetes support and education in the Look AHEAD study [6] into the meta-analysis. Even though diabetes education would encourage patients to lose weight and newly-diagnosed patients may make concerted efforts to do so, sustained large weight loss (> 10%) is hard to achieve with routine lifestyle changes. In real-word setting, intentional weigh loss attempt was generally set at 3–5% [41]. In the Look AHEAD trial, only 3.3% participants of the control group achieved a weight loss of more than 10%. Furthermore, studies have shown that following behavioural interventions, the body weight typically declines sharply during the first few months, followed by weight regain over the subsequent months to years [42]. In the ADDITION trial [12] that did not include any behavioural intervention, among participants who lost > 5% weight in the first year after diabetes diagnosis, 40% of them regained > 50% of the lost weight. Overall, large weight loss without behavioural interventions in studies included in the meta-analysis was indicative to be unintentional.

Unintentional weight loss may be idiopathic or occur due to various factors, such as cancer, drug interactions, depression, or other undiagnosed illnesses [43]. Major unintentional weight loss often serves as a marker of loss of physical reserve and disease severity in diabetic patients [13, 44]. In a large prospective trial of 2675 community-dwelling well-functioning older adults, T2DM was related to excessive loss of skeletal muscle and trunk fat mass [45]. In addition, older women with T2DM showed especially higher risk of loss of skeletal muscle mass compared to nondiabetic women. Malignancy is a common source of confounding for the relation between weight loss and mortality. To reduce the likelihood that weight loss was caused by malignant diseases, some studies [9, 10, 18] excluded participants with cancer, and some excluded deaths occurring in shortly after the assessment of weight change [8, 12]. In addition, no difference was observed in the cause of death across groups with different degrees of weight loss [11, 12].

### Interaction between weight loss and baseline BMI

Being overweight or obese is an established risk factor for cardiometabolic diseases. Weight management is highly recommended for these individuals. However, not only was weight loss found to be associated with increased mortality and CV outcomes, but several studies also reported a lower hazard of these major events in overweight or obese T2DM patients. In addition, patients who were underweight or had normal weight at the baseline had an increased risk of subsequent events. This phenomenon is referred to as the “obesity paradox”. In the ADDITION-Europe trial, large weight loss was associated with mortality among subjects with BMI < 30 kg/m^2^, whereas a null association was observed among subjects with BMI ≥ 30 kg/m^2^ [12]. Another study based on participant-reported weight loss found that overweight diabetic adults with an intention to lose weight had a reduced risk of mortality, regardless of whether they lost weight or not [29]. However, other three studies [8, 9, 19] did not find any significant influence of baseline weight on the association of weight loss with mortality or CVD events.

### Association between weight loss and outcomes among newly-diagnosed T2DM

For studies that included patients with established history of diabetes, the results were more consistent in terms of the relationship between weight loss and mortality. We analysed the results of studies that included only newly-diagnosed diabetes. Most of these studies [9, 11, 12, 23, 27] showed an association between weight loss and increased risk of mortality. In the Health, Aging, and Body Composition Study [45], newly-diagnosed T2DM patients had a remarkable decline in appendicular lean mass. This suggested that the loss of skeletal muscles was manifested in the early stages of diabetes. Weight reduction shortly after diagnosis of diabetes might be intentional as the patients are likely to be motivated by the new diagnosis. And moderate weight loss during the first year of diagnosis was observed to have reduced long-term CVD risk [7]. However, as demonstrated by the ADDITION-Europe study, substantial weight loss (> 10%) across a longer term after diagnosis was associated with a higher hazard of mortality.

### Interaction between weight loss and cardiovascular diseases

The results for the CV outcomes are more heterogeneous. In the Look AHEAD trial, participants who achieved ≥ 10% weight loss had 20% lower 10-year hazard of CVD. Since this study included only overweight or obese participants and half of them had intensive lifestyle intervention, the reduced hazard of CVD may have been partly attributable to the improvement of metabolic factors. This was demonstrated by another post-hoc analysis of this cohort [46]. Weight loss was associated with improvement in levels of HbA1c, systolic blood pressure, triglycerides, and HDL cholesterol, regardless of whether weight was regained or not. Even though data on the intention to lose weight is not available for most studies, participants that had adopted a healthy lifestyle had lower hazard of CVD and mortality [7, 11]. However, this protective effect may not necessarily be associated with the decreased weight. Several studies [47–49] have found that healthy behaviours are linked to improved cardiometabolic health with or without weight loss. In a prospective study of 1401 overweight diabetic patients, those with an intention to lose weight showed a reduced risk of mortality, independent of whether they lost weight or not [29]. Previous evidence has shown that a healthy lifestyle is important to maintain muscle mass and strength [13]. Previous studies [50, 51] in patients with coronary heart disease found that sustained physical activity, not weight loss was associated with reduced risk of mortality. These findings suggest that establishing a healthy lifestyle and sustained physical activity rather than focusing solely on weight loss may be more important [52, 53].

In the ADDITION trial, moderate weight loss at 1 year after diabetes diagnosis was associated with reduced hazard of 10-year CVD [7]. However, there was no conclusive evidence of the relation between maintenance of weight loss in the longer-term and CVD outcomes [12]. We pooled data of studies that investigated the relation between weight loss and a composite of CVD outcomes and CV death by weight loss magnitude. The results showed that participants with major weight loss, but not those with moderate weight loss, had an increased risk of composite of CVD events and CV death. However, this result was potentially biased due to the insufficient number of studies. For studies that were not included in the meta-analysis, most of them only found null associations between weight loss and CVD outcomes.

Since CV death is the most common cause of death in diabetic patients, the baseline CV risk profile of participants is likely to influence the association between weight loss and outcomes. The majority of these studies included participants with established CV risk factors. In the EXAMINE trial that included T2DM patients with a recent acute coronary syndrome, > 5% loss of weight was correlated with the increases in risk of subsequent CV events and mortality. Sensitivity analysis performed by excluding the EXAMINE trial yielded similar results. And within individual studies, similar adverse association with mortality was observed after adjusting for baseline CVD risk factors or after excluding participants with a history of CVD.

### Association between weight variability and outcomes

All five studies that investigated the association of weight variability with outcomes found an increased HR, even though they employed different indices for the measurement of weight fluctuation. The findings of the ADDITION-Europe trial are in line with these studies [12]. Among patients who lost > 5% weight at 1 year, those who regained weight at 5 years had higher hazard of mortality compared to those who did not regain weight. However, these findings are to some extent contrary to the results from the Look AHEAD trial in which large initial weight loss had improvement in CVD risk factors regardless of weight regain [46]. However, this analysis only included T2DM patients in the intensive lifestyle intervention arm. CVD risk factors may have improved due to other healthy lifestyle changes and not necessarily due to weight loss. Moreover, the study did not report follow-up data for mortality or CVD events. A community-dwelling population-based study suggested that large body weight fluctuation had protective effect on the incidence of DM in obese subjects, whereas the risk of incident DM in patients with BMI < 25 kg/m^2^ was increased [54].

## Limitations

Some limitations of this systematic review and meta-analysis should be considered while interpreting the results. First, there was considerable heterogeneity among the studies included in the meta-analysis. Several factors that may have contributed to the heterogeneity, including weight loss duration, duration of diabetes, severity of diabetes and underlying CVD risk. We performed sensitivity analysis and subgroup analysis aiming to reduce heterogeneity. However, the heterogeneity was primarily attributable to each of the individual study’s characteristics that could not be reduced effectively in our analyses. Therefore, we used the random effects model for meta-analysis, as this approach takes into account heterogeneity when computing the pooled effect estimate. Second, there was a difference in baseline age or initial BMI across the groups of weight change in most of the studies, which may have confounded the association between weight loss and outcomes. But almost all studies adjusted for baseline age and BMI when computing hazard ratio or relative risk ratio. And we only collected and pooled the rate ratios after full adjustment. Third, as we only pooled data of studies that categorized weight loss as 10% and 5%–10%, there were limited number of studies for the meta-analysis of the relation between weight loss and outcomes stratified by the magnitude of weight loss. Therefore, the results need to be interpreted with caution. Finally, most of the studies were observational or post-hoc analyses of clinical trials. Although major weight loss observed in most of studies was indicative to be unintentional, the effect of intentional weight loss needs to be evaluated in long-term intervention trials.

## Conclusions

Large weight loss and large fluctuations in weight are potential markers of increased risk of mortality and CV events in T2DM patients. Weight change as a global variable for physical condition may reflect the overall effect of multiple pathophysiologic processes, indicating disease progression and worsening health status. Management for T2DM patients should not focus solely on weight loss. Establishing healthy lifestyle and maintaining a stable weight may be more important.

## Supplementary Information

**Additional file 1. **Details of the search strategy for different databases.

**Additional file 2. **Additional Tables S1–S6 and Figures S1–S2.

## Data Availability

All data generated or analyzed during this study are presented in this article.

## Abbreviations

T2DM: Type 2 diabetes
CV: Cardiovascular
CVD: Cardiovascular diseases
HR: Hazard ratio
RR: Relative risks
ASV: Average successive variability
SD: Standard deviation
BMI: Body mass index
MI: Myocardial infarction
AF: Atrial fibrillation
Look AHEAD: Action for Health in Diabetes
EXAMINE: Cardiovascular outcomes study of alogliptin in patients with type 2 diabetes and acute coronary syndrome
ADDITION: Anglo–Danish–Dutch study of intensive treatment in people with screen-detected diabetes in primary care
