# Supplementary material for: Association of magnitude of weight loss and weight variability with mortality and major cardiovascular events among individuals with type 2 diabetes mellitus: a systematic review and meta-analysis
Source: Cardiovasc Diabetol. 2022 May 16;21:78. doi: 10.1186/s12933-022-01503-x (PMC9112517; doi:10.1186/s12933-022-01503-x)
Supplement: Supplementary file 1 — Additional file 1. Details of the search strategy for different databases. [file 12933_2022_1503_MOESM1_ESM.docx]

**Additional file 1:**

**Details of the search strategy for different databases**

Pubmed:

(((((((((("Diabetes Mellitus, Type 2"[Mesh]) OR (diabetes mellitus[Title/Abstract])) OR (diabetes[Title/Abstract])) OR (prediabetes[Title/Abstract])) OR (impaired fasting glucose[Title/Abstract])) OR (impaired glucose intolerance[Title/Abstract])) OR (borderline diabetes[Title/Abstract])) OR (blood glucose[Title/Abstract])) NOT ("Diabetes, Gestational"[Mesh] OR "Diabetes Mellitus, Type 1"[Mesh]))

AND

(((((((((((((((((((((((((((((((Mortality[MeSH Terms]) OR (cardiovascular event*[Title/Abstract])) OR (cardiovascular outcome*[Title/Abstract])) OR (Heart Failure[MeSH Terms])) OR (heart decompensation*[Title/Abstract])) OR (myocardial failure*[Title/Abstract])) OR (heart failure*[Title/Abstract])) OR (Atrial Fibrillation[MeSH Terms])) OR (atrial fibrillation*[Title/Abstract])) OR (Auricular Fibrillation*[Title/Abstract])) OR (Myocardial Infarction[MeSH Terms])) OR (myocardial infarct*[Title/Abstract])) OR (Cardiovascular Stroke*[Title/Abstract])) OR (Heart Attack*[Title/Abstract])) OR (major adverse cardiac event*[Title/Abstract])) OR (major adverse cardiovascular event*[Title/Abstract])) OR (major cardiovascular event*[Title/Abstract])) OR (major cardiac event*[Title/Abstract])) OR (MACE[Title/Abstract])) OR (ischemic stroke*[Title/Abstract])) OR (ischemic cerebrovascular accident*[Title/Abstract])) OR (ischaemic stroke*[Title/Abstract])) OR (ischaemic cerebrovascular accident*[Title/Abstract])) OR (myocardial ischaemia*[Title/Abstract])) OR (myocardial ischemia*[Title/Abstract])) OR (mortalit*[Title/Abstract])) OR (hazard ratio*[Title/Abstract])) OR (cardiovascular death[Title/Abstract])) OR (cardiac death[Title/Abstract])) OR (follow up[Title/Abstract])) OR (follow-up[Title/Abstract])))

AND

((((((((((((("Weight Loss"[Mesh]) OR "Body-Weight Trajectory"[Mesh]) OR "Body Weight Changes"[Mesh]) OR (weight change[Title/Abstract])) OR (weight loss[Title/Abstract])) OR (weight reduction*[Title/Abstract])) OR (losing weight[Title/Abstract])) OR (BMI change[Title/Abstract])) OR (body mass index change[Title/Abstract])) OR (body mass index loss[Title/Abstract])) OR (BMI loss[Title/Abstract])) OR (weight variability[Title/Abstract])) NOT ((((((((("Bariatric Surgery"[Mesh]) OR "Gastric Bypass"[Mesh]) OR (bariatric surg*[Title/Abstract])) OR (bariatric operat*[Title/Abstract])) OR (gastric bypass*[Title/Abstract])) OR (bariatric procedure*[Title/Abstract])) OR (obesity surg*[Title/Abstract])) OR (obesity operat*[Title/Abstract])) OR (metabolic surg*[Title/Abstract])))

Embase and Cochrane library databases were searched through Ovid using the above terms.
