# Supplementary material for: Association of magnitude of weight loss and weight variability with mortality and major cardiovascular events among individuals with type 2 diabetes mellitus: a systematic review and meta-analysis
Source: Cardiovasc Diabetol. 2022 May 16;21:78. doi: 10.1186/s12933-022-01503-x (PMC9112517; doi:10.1186/s12933-022-01503-x)
Supplement: Supplementary file 2 — Additional file 2. Additional Tables S1–S6 and Figures S1–S2. [file 12933_2022_1503_MOESM2_ESM.docx]

**Additional file 2: Additional tables and figures**

Table S1 Characteristics of studies included in the systematic review

| Study | Study name or dataset | Country/region | Study design | Year of enrollment | Diabetes duration | Baseline CVD excluded | Intervention | No. patients (% female) | Age (years) | Weight change measurement | Weight change interval | Follow-up | Event for analysis |
| --- | --- | --- | --- | --- | --- | --- | --- | --- | --- | --- | --- | --- | --- |
| Moazzeni et al, 2021 | Tehran Lipid and Glucose Study | Iran | Prospective | 1999-2002; 2002-2005 | NA | Yes | No | 763 (60.7) | Mean 53.6±11 | Percentage weight change from baseline (%) | 3 years | median 14.4 (IQR 12.1– 15.5) years | CVD, CHD |
| Strelitz et al, 2019 | ADDITION–Cambridge | UK | Prospective | 2002-2006 | Newly-diagnosed | No | multifactorial treatment and routine care | 725 (38.5) | Mean 61.1±7.1 | Percentage weight change from baseline (%) | 1 year | mean 9.8 years | All-cause mortality, CVD |
| Doehner et al, 2020 | ORIGIN trial | 40 countries | Prospective | 2003-2005 | Mean 5.0 years | No | insulin therapy, standard care, n-3 fatty acids or placebo | 12521(35) | Mean 63.5 | %weight change from baseline | 2 years | median 6.2 (IQR 5.8–6.7) | All-cause mortality, CV mortality, Primary composite endpoint, Expanded composite endpoint |
| Bodegard el al, 2013 | ROSE study | Sweden | Retrospective | 1999-2009 | Newly-diagnosed | Yes | No | 8486 (45.0) | Mean 60.0 (range 35–79) | BMI change from baseline (kg/m^2^) | 18 months | median 4.6 years （up to 9 years | CVD mortality, All-cause mortality |
| Xing et al, 2019 | ACCORD study | USA and Canada | Prospective | 2001- 2005 | Mean 10.75 ± 7.56 years. | No | intensive blood pressure, glycemic, lipid treatment, and standard care | 9372 (38.2) | Mean 62.7 | BMI change from baseline (kg/m^2^) | 2 years | mean 8.08 ± 3.00 years | All-cause mortality, Cardiac death, No-fatal death, No-cardiac death |
| Anyanwagu el al, 2018 | UK Primary Care | UK | Retrospective | 2007-2014 | Mean 4.3±4.9 years | Yes | insulin | 18814 (46.8) | Mean 61.5 ± 13.6 | Weight change from baseline (kg) | 1 year | mean 3.7±2.9 years | Mortality, Non-fatal MI, Non-fatal Stroke |
| Wedick et al, 2002 | Rancho Bernardo Study | USA | Prospective | 1972-1974 | NA | No | No | 230 (39.1) | Mean 59 | Weight change from baseline (pounds) | From Visit 1 (1972–1974) to Visit 2 (1984–1987) | up to 12 years | All-Cause Mortality |
| Gregg et al, 2004 | NHIS | USA | Retrospective | 1989 | Mean 10.5 years | No | No | 1401(57.6) | Mean 61.2 | Weight change from baseline (pounds) | 1 year | up to 9 years | All-cause mortality |
| Williamson et al, 2000 | Cancer Prevention Study I | USA | Retrospective | 1959-1960 | NA | No | No | 4951(49.4) | Mean 55 | Weight change (pounds) | NA | median 7.1 years | All-cause mortality |
| Polemiti et al, 2021 | EPIC-Potsdam study | Germany | Prospective | 1994-2009 | Newly-diagnosed | Yes | No | 1083 (45.8) | Median 60.4 (IQR 53.5–65.3) | relative BMI change per year | Mean 2.4 ± 0.55 years | median 10.8 (IQR (8.2–13.8) years | Macrovascular complications, Microvascular complications |
| Køster-Rasmussen et al, 2016 | DCGP | Denmark | Prospective | 1989-1992 | Newly- diagnosed | No | structured personal care or routine care | 419 (49.6) | Mean 62.1 | 1 kg of weight loss per year | Median(IQR): 6 （5.7–6.3） years | up to 13 years | All-cause mortality, Cardiovascular mortality, Cardiovascular morbidity |
| Nunes et al, 2017 | Optum Electronic Health Record database | USA | Retrospective | 2009.1-2014.12 | NA | No | sulfonylurea | 143635 (48.7) | NA | Weight change (%) | 1 year | up to 6 years | MI, stroke, CHF |
| Kocarnik et al, 2017 | Veterans Health Administration | USA | Prospective | 2004.1-2007.12 | Newly- diagnosed | No | Metformin, Glipizide, Glyburide, Rosiglitazone | 145198 (3) | Mean 65 | Weight change (%) | 1 year | 5 years | Mortality |
| Park et al, 2022 | KNHIS | Korea | Retrospective | 2007–2012 | NA | Yes | No | 1522241(36.3) | Mean 56.3 ± 12.0 | Weight change (%) | 2 years | Median 7.03 years (IQR 6.13–7.53) | MI, stroke, AF, HF, all-cause moratlity |
| Chan et al, 2021 | Chang Gung Research Database | Taiwan | Retrospective | 2001-2018 | mean 8.3 years | No | SGLT2i | 10237(42.4) | Mean 58.6 ± 11.5 | Weight change (%) | 3 months | Mean 1.5 ± 0.6 years | AF |
| Grundvold, et al, 2015 | ROSE study | Sweden | Retrospective | 2010 | Newly- diagnosed | Yes | No | 7169(45) | Mean 69 | Relative BMI change | 13.4 months | median 4.6years | AF |
| Lee HJ et al, 2020 | KNHIS | Korea | Retrospective | 2009-2010 | NA | No | No | 670797(35) | Mean 57.8 | Weight variability | 5 years | Mean 6.7 ± 1.3 years | AF |

Abbreviations: ADDITION: Anglo–Danish–Dutch Study of Intensive Treatment in People with Screen-Detected Diabetes in Primary Care; ORIGIN: Outcome Reduction with an Initial Glargine Intervention; ROSE: Retrospective Epidemiological Study to Investigate Outcome and Mortality with Glucose-lowering Drug Treatment in Primary Care; ACCORD: Action to Control Cardiovascular Risk in Diabetes; NHIS: National Health Interview Survey; EPIC: European Prospective Investigation into Cancer and Nutrition; DCGP: Diabetes Care in General Practice; CHD: coronary heart disease; MI: myocardial infarction; CHF: congestive heart failure; AF: atrial fibrillation; SGLT2i: sodium–glucose cotransporter 2 inhibitor.

Table S2 Quality assessment of the included studies using the Newcastle-Ottawa scale

| Study | Selection | | | |  | Outcome | | |  |
| --- | --- | --- | --- | --- | --- | --- | --- | --- | --- |
|  | Representativeness | Selection | Ascertainment | Outcome | Comparability | Assessment | Follow-up | Adequacy | Quality* |
| Lee et al, 2020 | * | * | * | * |  | * | * | * | 7 (good) |
| Kim et al, 2019 | * | * | * | * |  | * | * | * | 7 (good) |
| Gregg et al, 2016 | * | * | * | * |  | * | * | * | 7 (good) |
| Doehner et al, 2012 | * | * | * | * |  | * |  | * | 6 (fair) |
| Ferreira et al, 2021 |  | * | * | * |  | * |  | * | 5 (fair) |
| Hu et al, 2021 |  | * | * | * |  | * | * | * | 6 (fair) |
| Strelitz et al, 2021 | * | * | * | * |  | * | * | * | 7 (good) |
| Moazzeni et al, 2021 | * | * | * | * |  | * | * | * | 7 (good) |
| Strelitz et al, 2019 | * | * | * | * |  | * | * | * | 7 (good) |
| Doehner et al, 2020 | * | * | * | * |  | * | * | * | 7 (good) |
| Bodegard el al, 2013 | * | * | * | * | * | * | * | * | 8 (good) |
| Xing et al, 2019 | * | * | * | * |  | * | * | * | 7 (good) |
| Anyanwagu el al, 2018 |  | * | * | * | ** | * |  | * | 7 (good) |
| Wedick et al, 2002 |  | * | * | * |  | * | * | * | 6 (fair) |
| Gregg et al, 2004 | * | * |  |  | * | * | * | * | 6 (fair) |
| Williamson et al, 2000 | * | * |  | * |  | * | * | * | 6 (fair) |
| Polemiti et al, 2021 | * | * | * | * |  | * | * | * | 7 (good) |
| Køster-Rasmussen et al, 2016 |  | * | * | * |  | * | * | * | 6 (fair) |
| Nunes et al, 2017 | * | * |  | * |  | * | * | * | 6 (fair) |
| Kocarnik et al, 2017 |  | * | * | * |  | * | * | * | 6 (fair) |
| Park et al, 2022 | * | * | * | * |  | * | * | * | 7 (good) |
| Chan et al, 2021 |  | * | * | * |  | * |  | * | 5 (fair) |
| Grundvold, et al, 2015 | * | * | * | * |  | * | * | * | 7 (good) |
| Nam et al, 2021 | * | * | * | * |  | * | * | * | 7 (good) |
| Bangalore et al, 2018 | * | * | * | * |  | * | * | * | 7 (good) |
| Yeboah et al, 2019 | * | * | * | * |  | * | * | * | 7 (good) |
| Ceriello et al, 2021 | * | * | * | * |  | * | * | * | 7 (good) |
| Aucott et al, 2016 | * | * | * | * |  | * | * | * | 7 (good) |
| Kaze et al, 2022 | * | * | * | * |  | * | * | * | 7 (good) |
| Lee HJ et al, 2020 | * | * | * | * |  | * | * | * | 7 (good) |

Table S3 Outcomes of studies that reported association between weight loss and all-cause mortality

| First author | Study name | All-cause mortality, adjusted HR (95%CI) | | Reference |
| --- | --- | --- | --- | --- |
|  |  | Weight loss >10% | Weight loss 5%–10% |  |
| Lee et al, 2020^a^ | ADVANCE | **2.79 (2.10–3.71)** | **1.43 (1.16–1.76)** | Weight change ±4% |
| Kim et al, 2019 | KNHIS | **1.83 (1.59–2.12)** | **1.27 (1.15–1.41)** | Weight change±5% |
| Hu et al, 2021 | NHS and HPFS | **1.27 (1.11–1.46)** | 0.95 (0.83–1.07) | Weight change±5% |
| Strelitz et al, 2021 | ADDITION-Europe | **2.04 (1.17–3.35)** | 0.85 (0.47–1.54) | Weight change ±2% |
| Park et al, 2022 | KNHIS | **1.87(1.82–1.92)** | **1.26 (1.24–1.29)** | Weight change±5% |
|  |  | Weight loss >10% | Weight loss >5% |  |
| Doehner et al, 2012 | PROactive | **5.6 (3.96–7.91)** | **3.25 (2.51–4.21)** |  |
| Ferreira et al, 2021 | EXAMINE | **2.07 (1.31–3.25)** | **1.79 (1.33–2.42)** | Weight change±5% |
| Strelitz et al, 2019 | ADDITION–Cambridge |  | 1.12 (0.52–2.37) | Weight change ±2% |
| Nam et al, 2020 | KNHIS |  | **1.33 (1.28–1.38)** | Weight change±5% |
| Kocarnik et al, 2017 | Veterans Health Administration |  | **1.43 (1.33–1.53)^b^** | Weight change±5% |
| Doehner et al, 2020 | ORIGIN | Sustained weight loss | | Sustained stable weight or weight gain |
|  |  | **1.315 (1.182–1.463)** | |  |
| Bodegard el al, 2013 | ROSE study | BMI loss ≥ 1 kg/m^2^ | | BMI change ±1 kg/m^2^ |
|  |  | 1.06 (0.85–1.33) | |  |
| Xing et al, 2019 | ACCORD study | BMI loss > 5 kg/m^2^ | BMI loss 1.5–5kg/m^2^ | BMI change ±1.5 kg/m^2^ |
|  |  | **2.07 (1.68–2.55)** | 1.15 (1.00–1.32) |  |
| Anyanwagu el al, 2018 | UK Primary Care | Weight loss >5 kg | Weight loss 1.0–5.0 kg | Weight change ± 1 kg |
|  |  | 1.37 (1.01–1.89) | 1.14 (0.83–1.57) |  |
| Wedick et al, 2002 | Rancho Bernardo Study | Weight loss >10 pounds (Men) | Weight loss >10 pounds (Women) | Weight loss <10 pounds or weight gain |
|  |  | **3.85 (2.15–6.24)** | 1.58 (0.70–3.87) |  |
| Gregg et al, 2004 | NHIS | Unintentional weight loss | Intentional weight loss | Stable weight/weight gain (unintentional) |
|  |  | **1.58 (1.08–2.31)** | 0.83 (0.63–1.08) |  |
| Williamson et al, 2000 | Cancer Prevention Study I | Unintentional weight loss | Intentional weight loss | No change |
|  |  | 0.98 (0.85–1.13) | **0.75 (0.67–0.84)^c^** |  |
| Køster-Rasmussen et al, 2016 | DCGP | Unintentional weight loss | Intentional weight loss |  |
|  |  | **1.21 (1.03–1.41)** | 1.20 (0.97–1.50) |  |

a: moderate weight loss was defined as 4%–10%; b: the given result is odds ratio; c: the given result is relative risk.

Abbreviations: ADVANCE: The Action in Diabetes and Vascular disease: preterAx and diamicroN-MR Controlled Evaluation; KNHIS: Korean National Health Insurance System; PROactive, PROspective pioglitAzone Clinical Trial In macroVascular Events; EXAMINE: Cardiovascular Outcomes Study of Alogliptin in Patients With Type 2 Diabetes and Acute Coronary Syndrome; NHS: Nurses’ Health Study; HFPS: Health Professionals Follow-up Study, other abbreviation as in Table S2;

Table S4 Outcomes of studies that reported association between weight loss and cardiovascular endpoints

| First author | Study | Cardiovascular events, adjusted HR (95%CI) | | | Reference |
| --- | --- | --- | --- | --- | --- |
|  |  |  | > 10% | 5%–10% |  |
| Lee et al, 2020 | ADVANCE^a^ | composite | **1.75 (1.26–2.44)** | 1.08 (0.87–1.34) | Weight change ±4% |
|  |  | CV mortality | **2.76 (1.87–4.09)** | 1.06 (0.77–1.46) |  |
| Gregg et al, 2016 | Look AHEAD (control group) | Composite of CV outcomes | 0.71 (0.33–1.51) | 1.26 (0.88–1.79) | Weight loss <2% or weight gain |
| Hu et al, 2021 | NHS | CV mortality | **1.46 (1.15–1.86)** | 1.04 (0.83–1.30) | Weight change ±5% |
| Kim et al, 2019 | KNHIS | MI | 1.08 (0.85–1.38) | 1.10 (0.96–1.27) | Weight change ±5% |
|  |  | Stroke | 1.08 (0.87–1.34) | 0.97 (0.85–1.11) |  |
| Strelitzet al, 2021 | ADDITION-Europe | Composite of CV outcomes | 1.50 (0.85–2.66) | 1.05 (0.62–1.80) | Weight change ±2% |
| Park et al, 2022 | KNHIS | MI | 1.24 (1.17–1.32) | 1.11 (1.08–1.15) | Weight change ±5% |
|  |  | stroke | 1.20 (1.14–1.26) | 1.09 (1.06–1.12) |  |
|  |  | AF | 1.24 (1.17–1.31) | 1.09 (1.06–1.13) |  |
|  |  | HF | 1.41 (1.36–1.47) | 1.14 (1.11–1.16) |  |
|  |  |  | > 10% | > 5% |  |
| Ferreira et al, 2021 | EXAMINE | Composite of CV outcomes | **2.14 (1.15–1.86)** | **1.67(1.32–2.10)** | Weight change ±5% |
|  |  | CV mortality | **1.89 (1.10–3.26)** | **1.84 (1.30–2.61)** |  |
| Moazzeni et al, 2021 | Tehran Lipid and Glucose Study | Composite of CV outcomes |  | 1.11 (0.79–1.56) | Weigh change ±3% |
| Strelitzet al, 2019 | ADDITION–Cambridge | Composite of CV outcomes |  | **0.52 (0.32–0.86)** | Weight change ±2% |
| Nam et al, 2020 | KNHIS | MI |  | **1.18 (1.13–1.24)** | Weight change ±5% |
|  |  | Stroke |  | **1.09 (1.03–1.14)** |  |
| Nunes et al, 2017 | Optum Electronic Health Record database | AMI |  | **1.39 (1.21-1.60)** | Weight change ±0.5% |
|  |  | Stroke |  | **1.50 (1.33-1.70)** |  |
|  |  | CHF |  | **1.64 (1.50-1.79)** |  |
| Chan et al, 2021 | Chang Gung Research Database | AF |  | **0.39 (0.22–0.68)** | No weight loss |
| Doehner et al, 2020 | ORIGIN |  | Sustained weight loss |  | Sustained stable weight or weight gain |
|  |  | Composite of CV outcomes | 1.049 (0.942–1.168) |  |  |
|  |  | CV mortality | **1.175 (1.020–1.353)** |  |  |
| Bodegard el al, 2013 | ROSE study |  | BMI loss ≥1 kg/m^2^ |  | BMI change between ±1 kg/m^2^ |
|  |  | CV mortality | 1.06 (0.76–1.48) |  |  |
| Xing et al, 2019 | ACCORD study |  | BMI change >5 kg/m^2^ | BMI loss 1.5–5kg/m^2^ | BMI change ±1.5 kg/m^2^ |
|  |  | CV mortality | 1.29 (0.828–2.00) | 1.05 (0.822–1.35) |  |
|  |  | Non-fatal MI | 1.03 (0.717–1.48) | 1.02 (0.849–1.24) |  |
| Anyanwagu el al, 2018 | UK Primary Care |  | Weight loss >5kg | Weight loss 1.0–5.0kg | Weight change ±1kg |
|  |  | Composite of CV outcomes | **1.31 (1.02–1.68)** | 1.15 (0.90–1.47) |  |
|  |  | Non-fatal MI | 5.06 (0.68–37.67) | 4.26 (0.58–31.57) |  |
|  |  | Non-fatal stroke | 1.15 (0.77–1.71) | 1.10 (0.74–1.63) |  |
| Williamson et al, 2000 | Cancer Prevention Study I |  | Unintentional loss | Intentional loss | No change |
|  |  | Death from CVD and diabetes | 0.98 (0.83–1.15) | **0.72 (0.63–0.82)**^b^ |  |
| Polemiti et al, 2021 | EPIC-Potsdam study |  | Relative annual BMI loss > 1% |  | Relative annual BMI between ±1% |
|  |  | Composite of MI and stroke | 1.04 (0.62–1.74) |  |  |
| Køster-Rasmussen et al, 2016 | DCGP |  | Intention to lose weight | Intention to maintain weight |  |
|  |  | CV mortality | 1.06 (0.79–1.42) | 0.95 (0.77–1.17) |  |
|  |  | Composite of CV outcomes | 1.06 (0.79–1.42) | 0.95 (0.77–1.17) |  |
| Grundvold et al, 2015 | ROSE study |  | Relative weight loss >1 BMI unit |  |  |
|  |  | AF | 1.06 (0.81–1.38) |  |  |

a: moderate weight loss was defined as 4%–10%; b: the given result is relative risk.

Abbreviations: Look AHEAD: Action for Health in Diabetes; Other abbreviations as in Table S1 and Table S2.

Table S5 Adjusted confounders in each study that included in the meta-analysis

| First author | Study | Confounder adjusted |
| --- | --- | --- |
| Lee et al, 2020 | ADVANCE | age, sex, country grouping, baseline HbA1c, baseline systolic blood pressure, history of macrovascular disease at baseline, history of microvascular disease at baseline, randomized glucose treatment assignment, randomized blood pressure treatment assignment, baseline smoking status (current/former/never), baseline statin use, and diabetes medication use (metformin/TZDs/sulfonylureas/insulin) at year 2 |
| Kim et al, 2019 | KNHIS | Age, sex, smoking, alcohol consumption, regular exercise, income status, baseline FBG, dyslipidemia, hypertension, waist circumference, baseline use of insulin, and estimated glomerular filtration rate. |
| Gregg et al, 2016 | Look AHEAD | sex, age, baseline weight (from weight-change models), baseline fitness (from fitness-change models), history of cardiovascular disease, insulin use, diabetes duration, smoking status, LDL cholesterol, systolic blood pressure, and diastolic blood pressure. |
| Doehner et al, 2012 | PROactive | age, creatinine, LDL, HbA1c, previous MI, previous stroke, previous PCI or CABG, peripheral obstructive artery disease, smoking status, Insulin use, diuretic use, and statin use |
| Moazzeni et al, 2021 | Tehran Lipid and Glucose Study | age and sex, BMI, educational level, current smoking (at first follow-up), GLD use (at baseline or first follow-up), family history of premature CVD, hypertension, hypercholesterolemia, CKD, and FPG |
| Ferreira et al, 2021 | EXAMINE trial | treatment (alogliptin or placebo), diabetes duration, insulin, metformin, statins, current smoking, heart failure history, anemia, hypertension, BMI, eGFR, and diuretics. |
| Hu et al, 2021 | NHS and HPFS | age, sex, ethnicity, history of hypertension, current antidiabetic medication use, current antihypertensive medication use, hypercholesterolemia, current anti-hypercholesterolemia medication use in the questionnaire cycle right after diabetes diagnosis, family history of diabetes, family history of myocardial infarction, current aspirin use, current multivitamin use, diabetes duration, and lifestyle score before diabetes diagnosis. |
| Strelitz et al, 2021 | ADDITION-Europe | trial arm, study center, sex, age at diabetes diagnosis, weight at baseline, smoking status at baseline, CVD event within 5 years following diabetes diagnosis, anti-hypertensive medication use prior to diabetes diagnosis, lipid-lowering medication use prior to diabetes diagnosis, changes in each of anti-hypertensive, lipid-lowering, and glucose-lowering medication use between baseline and 5 years, and age left full-time education. |
| Strelitz et al, 2019 | ADDITION-Cambridge | age, sex, baseline SES, baseline BMI, smoking at 1 year, use of antihypertensive, lipid- or glucose-lowering medication at 1 year, and trial arm |
| Nam et al, 2021 | KNHIS | age, sex, smoking status, alcohol consumption, physical activity, income, hypertension, dyslipidemia, chronic kidney disease, insulin use, number of oral antidiabetic agents used, and baseline BMI. |
| Bangalore et al, 2018 | CARDS, ASPEN, and TNT trial | age, sex, race, hypertension, and smoking; mean weight and weight change (taking directionality into account); study treatment; baseline levels of LDL cholesterol, HDL cholesterol, total cholesterol, and triglycerides; chronic kidney disease and study; and time between initial and final weight measurements. |
| Yeboah et al, 2019 | ACCORD study | age, gender, race, arm of the trial, baseline BMI, statin use, GFR, mean SBP, mean DBP, mean LDL, mean HDL, mean HBA1C, years of diabetes, cigarrete smoking status, antihypertensive medication use, baseline cardiovascular disease status, time between initial and final weight measurement. |
| Ceriello et al, 2021 | Swedish National  Diabetes Register | age, gender, duration of diabetes, body weight, smoking, values of HbA1c, systolic and diastolic blood pressure, total cholesterol, HDL, LDL, triglycerides, albuminuria, eGFR, retinopathy, treatment for diabetes, hypertension, dyslipidemia, and aspirin use |
| Aucott et al, 2016 | SCI-DC | age, BMI, sex, smoking status, deprivation, weight change patterns and antidiabetic medication regimes |
| Kaze et al, 2022 | Look AHEAD | age, sex, race, ethnicity, current smoking, alcohol drinking, use of antihypertensive medications, mean systolic blood pressure, mean ratio of total to high-density lipoprotein cholesterol, mean hemoglobin A1c level, estimated glomerular filtration rate, and duration of diabetes, mean BMI |

Table S6 Adjusted confounders in each study that included in the systematic review

| First author | Study | Confounder adjusted |
| --- | --- | --- |
| Doehner et al, 2020 | ORIGIN trial | Age, sex, smoking status, previous CV events, duration of DM, waist circumference, systolic blood pressure, medication with ACE or ARB, beta-blocker medication, statin, LDL level, HbA1c level, eGFR, allocation to treatment arm glargine, and allocation to treatment arm n-3 fatty acid (full adjustment model). Previous CV events were defined according to the ORIGIN protocol as a history of MI, stroke, or revascularization. |
| Bodegard el al, 2013 | ROSE study | Age, gender, BMI at baseline and previous angina pectoris, education, marital status and use of glucose-lowering drugs. |
| Xing et al, 2019 | ACCORD study | Age, sex, smoking status, hypertension, hyperlipidemia, previous cardiovascular events, proteinuria, depression, HbA1c level, and fasting blood glucose level. |
| Anyanwagu el al, 2018 | UK Primary Care | Age, diabetes duration diastolic and systolic blood pressure, height, weight, albumin, glomerular filtration rate, gender, smoking status, alcohol status, lipid profile, Townsend Deprivation score, number of glucose-lowering therapies, Lipid-lowering therapies, antihypertensives, heart failure and peripheral artery disease |
| Wedick et al, 2002 | Rancho Bernardo Study | Age, current or recent smoking, and exercising less than 10 years earlier. |
| Gregg et al, 2004 | NHIS | Age, race, sex, education, smoking, “initial” BMI (BMI before weight change), measures of health status (self-rated health, functional limitations, heart disease, stroke, retinopathy, and neuropathy), measures of health care use (past year hospitalizations and doctor visits), insulin use, and years since diabetes diagnosis |
| Williamson et al, 2000 | Cancer Prevention Study I | age, sex, race, smoking, initial BMI, education, drinking, physical activity, disease history, and current signs and symptoms |
| Polemiti et al, 2021 | EPIC-Potsdam study | Age, sex, education, smoking status, smoking duration, physical activity, alcohol consumption, MedPyr score, family history of diabetes, myocardial infarction and stroke, hypertension and dyslipidemia |
| Køster-Rasmussen et al, 2016 | Diabetes Care in General Practice | age, sex, education, BMI at diagnosis, change in smoking, change in physical activity, change in medication, and the Charlson comorbidity 6-year score |
| Nunes et al, 2017 | Optum Electronic Health Record database. | Age, gender, prior CVD, baseline endocrinology visits, baseline cardiology visits, HbA1c, OAD categories and insulin prescriptions |
| Kocarnik et al, 2017 | Veterans Health Administration | Age, co-morbidities, alcohol consumption, tobacco use, use of other medications, baseline HDL concentration, baseline LDL concentration, baseline serum creatinine concentration, A1c level at the time of OHA initiation, and A1c level one year after OHA initiation |
| Park et al, 2022 | KNHIS | age, sex, previous history of hypertension, dyslipidemia, cancer, hyperthyroidism, chronic kidney disease, peripheral artery disease, chronic obstructive pulmonary disease, income level, smoking status, drinking habit, regular physical activity, insulin medication, use of oral hypoglycemic agents, and body mass index ≥ 25 kg/m |
| Chan et al, 2021 | Chang Gung Research Database | age, sex, different SGLT2i drugs and dosage, baseline comorbidities, HbA1c, eGFR, and use of antiplatelet therapy, statin, angiotensin system inhibitor, and all anti‑hypoglycemic agents |
| Grundvold et al, 2015 | ROSE study | age, gender, body mass index at baseline and previous angina pectoris, systolic blood pressure |
| Lee HJ et al, 2020 | KNHIS | baseline body mass index, age, sex, smoking, drinking, exercise, low income, hypertension, dyslipidemia, number of oral anti‑diabetic medication, insulin use, duration of diabetes, and fasting glucose |


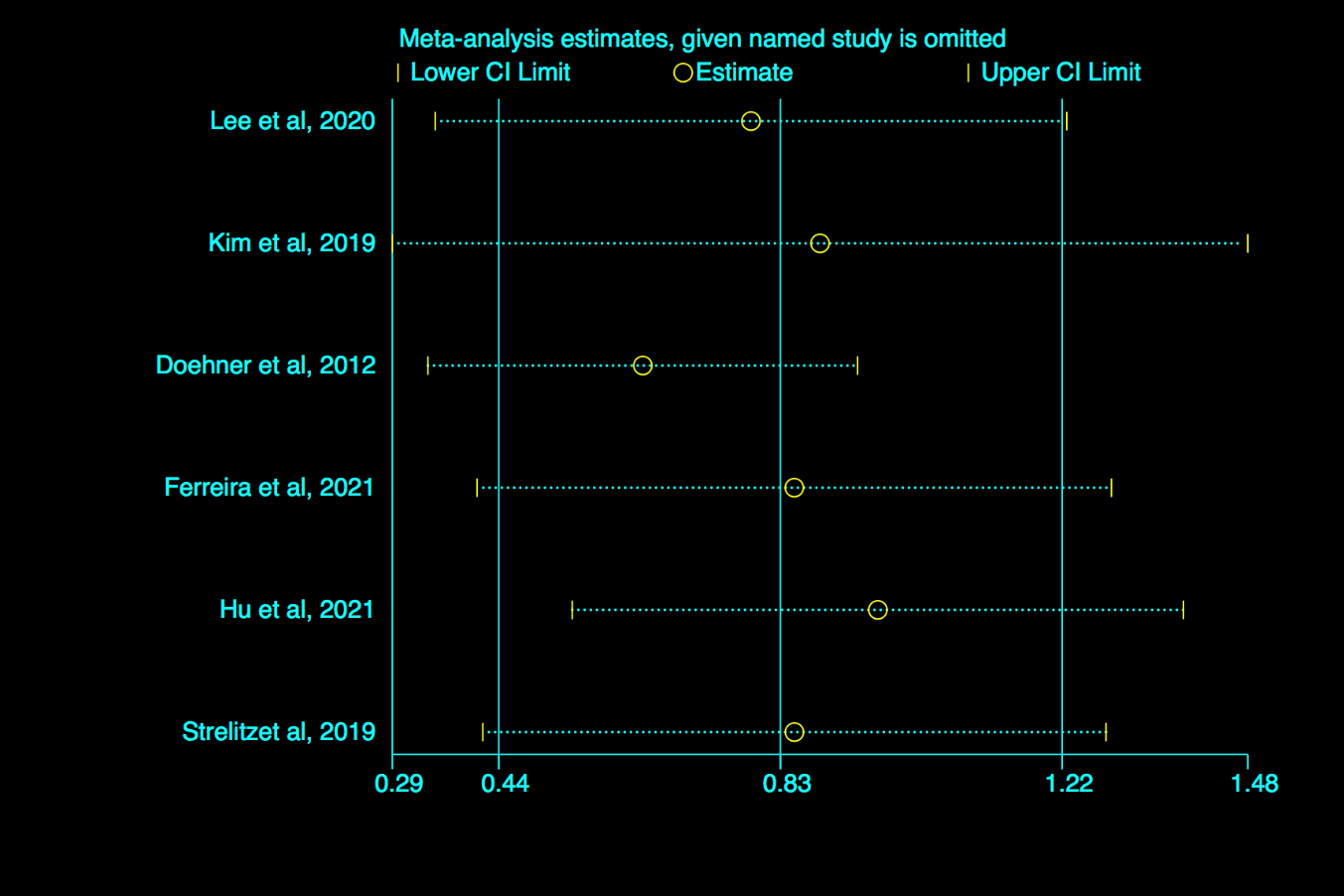


Figure S1 Sensitivity analysis of studies that investigated association between large weight loss and all-cause mortality


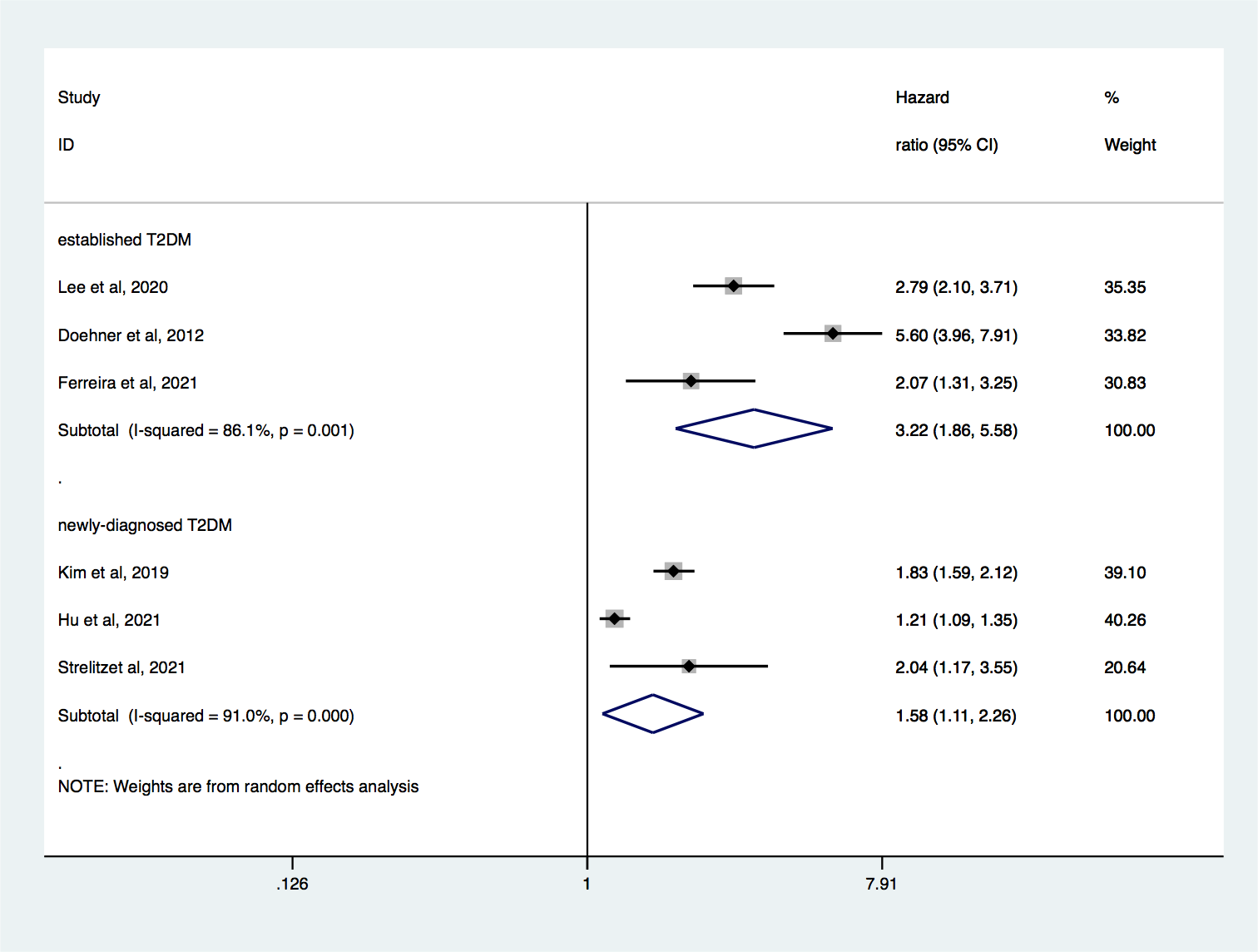


Figure S2 Forest plot of hazard ratios of all-cause mortality for large weight loss in participants with established T2DM and newly-diagnosed T2DM.


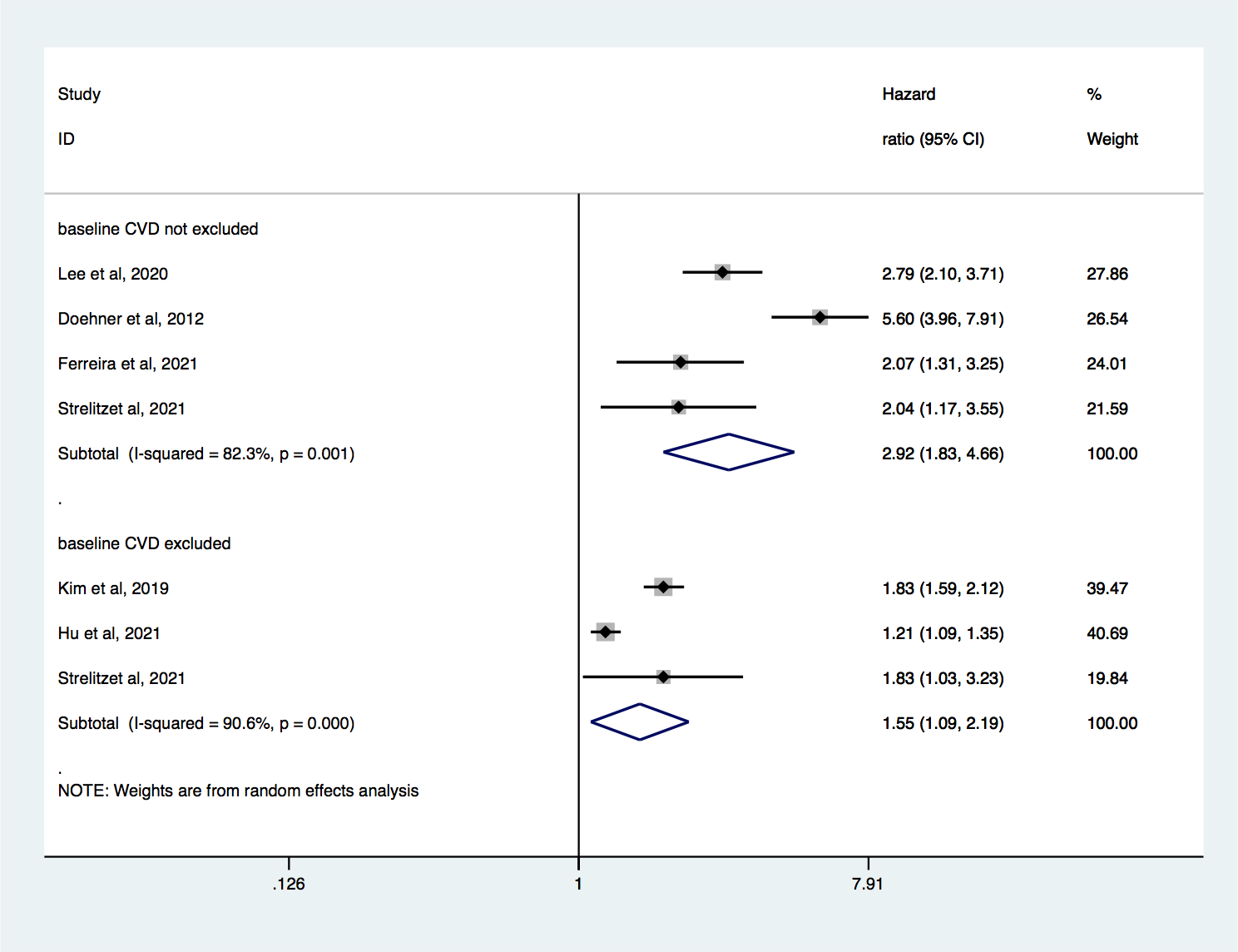


Figure S3 Forest plot of hazard ratios of all-cause mortality for large weight loss in participants without baseline cardiovascular diseases.
